# Analysis of Liquid Sweat Transport in Underwear Combined with Multilayer Fabric Assemblies for Firefighter Outfits

**DOI:** 10.3390/ma17235920

**Published:** 2024-12-03

**Authors:** Małgorzata Matusiak, Otgonsuren Sukhbat

**Affiliations:** Lodz University of Technology, Faculty of Material Technologies and Textile Design, Institute of Architecture of Textiles, 90-924 Lodz, Poland; otgoo999@gmail.com

**Keywords:** liquid transport, comfort, underwear, knitted fabrics, firefighters protective clothing, Moisture Management Tester

## Abstract

A firefighter’s outfit consists of several layers with distinct properties and functions. These layers serve as barriers against external hazards but also impede the transport of sweat generated by the human body. As a result, sweat vapor often fails to transfer effectively from the body through the firefighter’s protective clothing (FPC) to the environment. This can lead to sweat condensation on the firefighter’s skin, causing discomfort. To enhance the physiological comfort of firefighters during firefighting and other rescue operations, it is essential to consider the transport of condensed sweat within the multilayer textile system comprising both the underwear and the FPC. In this study, 16 assembly variants were tested, combining four types of knitted fabrics for underwear with four types of multilayer textile sets designed for FPC. The liquid moisture transport properties of these assemblies were evaluated using the Moisture Management Tester (MMT290), an innovative instrument manufactured by SDL Atlas. The results demonstrated that the knitted fabrics effectively transport liquid sweat, whereas in the case of multilayer textile sets for FPC, liquid sweat transport is primarily confined to the inner layer adjacent to the skin. Furthermore, the findings indicate that by selecting an appropriate combination of knitted fabric for underwear and the inner layer of the FPC, it is possible to optimize liquid moisture transport in a firefighter’s outfit.

## 1. Introduction

The job of firefighters is among the most dangerous professions. Their duties extend beyond firefighting to include various rescue operations such as in roadway accidents, aircraft fires, emergency medical interventions, and flood response. Each type of emergency operation presents entirely different risks. A firefighter’s full outfit during operations can consist of up to six layers, including underwear, which serve as barriers against hazardous external factors. Firefighter underwear is typically made from knitted fabrics based on cotton fibers. However, blends containing special fibers with non-flammable and flame-retardant properties are also used, though less frequently than cotton-based fabrics. In action, firefighters wear underwear in combination with firefighter protective clothing (FPC), which generally comprises three main layers: the outer shell, a moisture barrier, and a thermal barrier [1,2,3,4,5]. The outer shell is made from non-flammable fabric designed to provide protection against open flames and mechanical impacts. It also offers some resistance to toxic substances. The moisture barrier, made of polymer materials, protects against moisture and wind, while the thermal barrier, composed of materials with low thermal conductivity, shields against heat exposure. Although the FPC is commonly described as a three-layer assembly in the scientific literature, the actual number of individual materials used is greater. The outer shell, moisture barrier, and thermal barrier are the primary components, but additional protective features are integrated. For instance, the outer shell is often treated with special impregnations for enhanced performance, while the nonwoven thermal insulation is safeguarded by a lining, forming a two-layer structure [6].

The FPC acts as a barrier against hazardous factors encountered during firefighting and other rescue operations. However, it also impedes the transfer of metabolic heat and sweat produced by the human body. Sweat transfer is addressed in standards that define the requirements for firefighter protective clothing. These standards, however, primarily consider water-vapor resistance. Water-vapor transfer through textile materials occurs via the diffusion of water-vapor molecules through the pores in the fabric. Consequently, the water-vapor permeability of fabrics largely depends on their structure, particularly their porosity. According to the standards, the water-vapor resistance of FPC should not exceed 45 m^2^Pa/W. This parameter represents the FPC’s ability to transfer sweat in the form of vapor. The EN 469:2021 standard [7], however, does not include requirements for liquid moisture transport. Similarly, there are no specific guidelines concerning the transport of liquid sweat for firefighter underwear. Underwear, being the layer that adheres directly to the skin, is the first to come into contact with sweat condensed on the skin. Research to date has seldom addressed the issue of sweat condensation on the firefighter’s skin or the transport of liquid sweat through FPC [3,8,9,10]. Yet, the transport of condensed (liquid) sweat significantly affects the thermo-physiological comfort of firefighters during strenuous rescue operations. In high-stress or hot conditions, intense activity leads to excessive sweating. Some of this sweat cannot evaporate due to the high water-vapor resistance of the multilayer protective clothing, resulting in condensation on the skin’s surface. In such cases, it is essential for liquid sweat to be transported through the underwear and FPC layers to the outermost layer of the outfit, where it can then evaporate into the environment. The transport of liquid moisture through textiles is governed by two key processes: wetting and wicking. Wetting refers to the replacement of a solid–air interface with a solid–liquid interface, allowing the liquid to spread across the fabric surface. This process is determined by the surface energies of the solid and liquid involved, with wettability indicating the material’s potential to interact with liquids. Wicking, on the other hand, is the spontaneous flow of liquid within fabrics, driven by capillary forces. It involves the movement of liquid into the capillary spaces within the fabric, such as those between fibers in yarns and between yarns in fabrics. Smaller capillaries generate stronger capillary forces, meaning that finer fibers and yarns create smaller gaps in the fabric structure, thereby enhancing moisture transport via the wicking mechanism.

The type of raw material—specifically the fibers—also significantly affects liquid moisture transport in fabrics. A key factor is the hydrophilicity of the fibrous material. Hydrophilic fibers, such as cotton, readily absorb moisture. The negatively charged hydroxyl (OH) groups in cellulose, the main component of cotton fibers, attract water molecules, binding water within the fibers. This absorption limits the transport of liquid by capillary forces in cotton fabrics. In contrast, fabrics made from hydrophobic fibers, such as polyester, do not absorb liquid water. This characteristic facilitates the transport of liquid moisture through capillary forces and allows for rapid evaporation. Since the liquid water remains on the fiber surface rather than being absorbed, it is more readily transferred and evaporated.

Given the presence of liquid sweat on a firefighter’s skin, it is essential to consider liquid moisture transport when selecting both the underwear and the materials for FPC. Increased humidity in the multilayer materials of the FPC, resulting from sweat produced by the human body, reduces the protective performance of the clothing. This reduction occurs because liquid sweat retained within the materials raises their thermal conductivity, diminishing the FPC’s ability to provide thermal insulation.

Numerous research studies focus on the investigation and optimization of materials used in FPC and its multilayer assemblies [11,12,13,14,15]. In contrast, studies examining the role of underwear when worn beneath FPC are relatively rare. However, as demonstrated in many studies, clothing worn close to the body, such as underwear, significantly affects the user’s thermal sensations. Discomfort associated with wearing protective clothing can be greatly alleviated by selecting appropriate underwear. During strenuous activities, such as a firefighter’s mission, sweat produced by the body must be efficiently transferred through the clothing assembly to the surrounding air. If this transfer does not occur, heat and moisture accumulate within the clothing, leading to a decrease in the firefighter’s performance [16,17]. As the body’s “second skin”, underwear serves the dual functions of providing protection and ventilation. For this reason, the choice of material for firefighter underwear is particularly important.

In the underwear market, cotton and viscose fibers are the primary raw materials, both of which have a similar chemical composition based on cellulose [18,19]. Cotton/polyester blends are also commonly used in the knitted fabrics for t-shirts. These blends are stronger than pure cotton fabrics and offer a broader range of textures. Since underwear is in direct contact with the human body, it plays a crucial role in maintaining the body’s thermal balance during fluctuations in physical activity. Additionally, it helps regulate perspiration, manage moisture, and maintain the microclimate around the skin, which is influenced by body heat. This makes the choice of materials for underwear essential for comfort and performance.

Underwear specifically designed for firefighters is now becoming available in commercial markets. This functional underwear is typically fire-resistant and intended to be used in combination with outer fire-resistant protective garments as a secondary protective layer. It offers physiological and wearing comfort, a soft touch, and efficient moisture transfer, achieved through the appropriate selection of raw materials. Such underwear is commonly made from modacrylic/cotton blends [20]. It is crucial that underwear, together with the FPC, forms an optimal assembly that balances both protection and comfort. For this reason, in addition to testing the individual components of a firefighter’s outfit, it is essential—or at least advisable—to evaluate the performance of the entire assembly of textile materials that make up the outfit.

It should be noted that the combination and arrangement of fabrics in a firefighter’s protective outfit can significantly influence the garment’s fit, flexibility, and mobility. First and foremost, the fit of the garment depends on the pattern construction and garment size, which should align with the anthropometric characteristics of the firefighter’s body. In the case of underwear, which is typically made of thin, stretchable knitted fabrics, it fits the human body very well without restricting mobility. On the other hand, the situation is quite different for the firefighter’s special garments. These garments are composed of several layers made from thick, stiff textile materials. Each layer must be in the correct order to perform its intended function. The thermal layer is designed with a predetermined thickness to ensure thermal insulation (thermal resistance), while the outer layer protects against external factors such as flames and fluids. Typically, firefighter garments are thick and stiff, with air spaces between the layers. However, these air spaces and their thickness are not permanent because the textile materials used are generally flexible. During movement, the entire garment, along with its individual layers, shifts. This results in changes in the distance between the layers and the garment’s fit to the body, particularly the interaction between the protective clothing and the underwear worn beneath it. The stiffness and thickness of the clothing can negatively impact comfort, especially the freedom of movement. Nonetheless, the appropriate stiffness and thickness of firefighter protective clothing are necessary to ensure the safety of the firefighter in action. It is well-known that the protective properties of firefighter clothing often conflict with the comfort factors, particularly thermo-physiological comfort and fit. Despite this, ensuring the safety of the firefighter is the foremost priority, outweighing comfort considerations. The application of different knitted underwear in combination with various multilayer FPC sets does not affect the fit, flexibility, or mobility of the overall protective ensemble.

The aim of the presented work was to investigate knitted fabrics for underwear that can be used by firefighters, specifically in terms of liquid moisture management. The knitted fabrics were tested both individually as a single layer and in combination with multi-layer textile sets designed for the FPC. Measurements were conducted using the Moisture Management Tester by SDL Atlas [21,22,23,24,25,26,27].

## 2. Materials and Methods

Four variants of knitted fabrics intended for underwear were the subjects of the investigations. These were: single jersey cotton fabric, pique cotton fabric with 3% elastane, single jersey cotton/polyester knitted fabric, and a special knitted fabric designed for firefighters. The latter fabric was composed of a blend of cotton, modacrylic, and polyamide, with the addition of antistatic fiber and elastane. The fabrics F1–F3 underwent the same finishing process, which included washing, dyeing, and stabilization. The finishing of the F4 fabric involved washing and stabilization only. This fabric is made of black and white fibers, creating a grey mélange effect, which is why it was not dyed. The basic properties of the investigated knitted fabrics are presented in Table 1.

The knitted fabrics were measured individually as a single-layer material and then in combination with the multilayer textile sets intended for the FPC. These multilayer textile sets were sourced from the Polish industry and are commercially available packages specifically developed for the FPC. The manufacturers of the investigated materials confirmed that their protective properties have been tested and certified according to the relevant standards. In the context of this work, only the liquid moisture transport properties of the multilayer sets were measured and analyzed. The characteristics of the multilayer textile sets used in the investigation are presented in Table 2. The data provided in the table were supplied by the manufacturers. However, more detailed data on the individual components could not be disclosed due to market competitiveness.

In all cases, the lining is made of woven fabrics with a plain weave. The structure of all the lining fabrics is similar. The warp and weft densities are as follows: S1: warp density—24/cm, weft density—24/cm; S2 and S3: warp and weft density—22/cm; and S4: warp density—22/cm, weft density—20/cm. As shown in Table 2, in the S1 multilayer set, the lining is made of cotton woven fabric with a fire-retardant finish.

**Figure 1:**
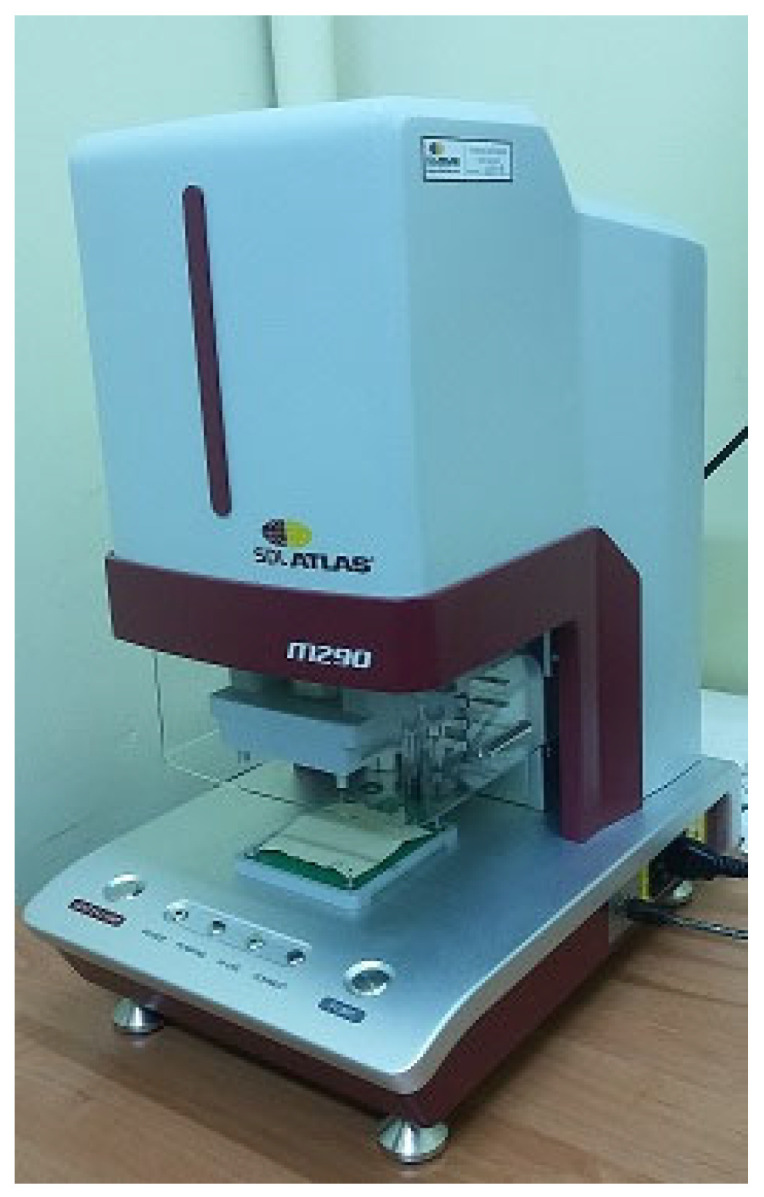
Moisture Management Tester MMT M290.

To measure the liquid moisture transport of the knitted fabrics, the Moisture Management Tester [21,22,23,24,25,26,27] by SDL Atlas (Rock Hill, SC, USA) was used (Figure 1). This instrument allows for the measurement of textile materials’ liquid transport properties in the following aspects [21,22]:

- Time of moisture absorption—determined for both surfaces of the measured specimen: inner and outer,
- Transfer of liquid moisture in one direction: from the inner surface to the outer,
- Speed of spreading liquid moisture on both fabric surfaces.

**Figure 2:**
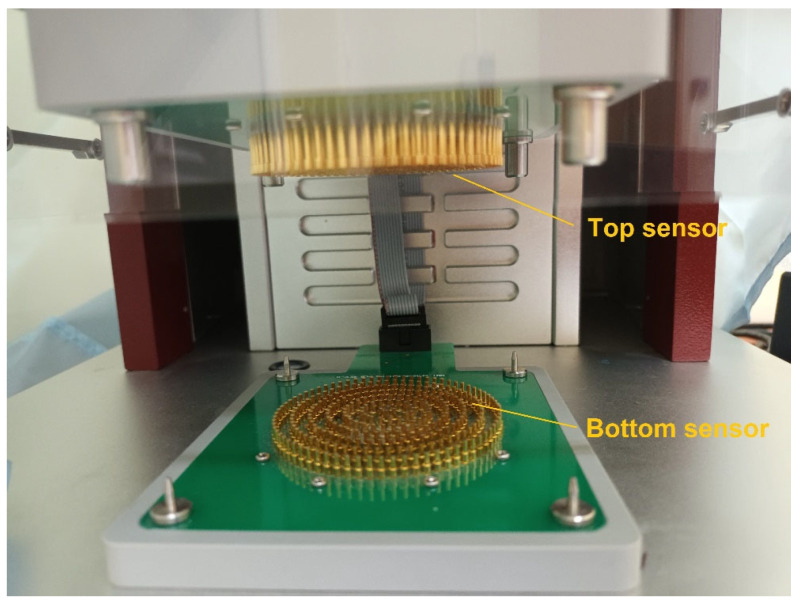
Top and bottom sensors of the MMT M290.

This device is equipped with two electric sensors: an upper sensor and a lower sensor (Figure 2). The electric signal generated by the sensors is converted into moisture data. During testing, the measured specimen is placed between the sensors, as shown below.

The measurement is carried out following the procedure described in the AATCC standard and the device’s instruction manual [21,22]. A precisely determined quantity of testing solution is dispensed drop by drop onto the upper surface of the measured sample. The upper surface represents the side of the material that is closest to the user’s body during clothing use. The liquid is then transferred onto the measured sample in three directions:

- Spreading centrally on the upper surface,
- Transfer from the upper to the bottom surface,
- Spreading on the bottom surface of the measured sample.

The measurements were performed according to the standardized method [21,22].

The results of the measurements were analyzed statistically using multifactor ANOVA. In the statistical analysis, the variant of knitted fabrics and the variant of multilayer textile sets were treated as independent variables, while the specific parameters from the MMT were analyzed separately as dependent variables.

Statistical significance was assessed at a significance level of 0.05.

## 3. Results and Discussion

The MMT provides 10 parameters that characterize the liquid moisture transport in the measured specimen. These parameters are as follows: wetting time of top (WTT) and bottom (WTB) surfaces, absorption rate of top (TAR) and bottom (BAR) surfaces, maximum wetted radius on top (MWRT) and bottom (MWRB) surfaces, spreading speed on top (SST) and bottom (SSB) surfaces, accumulative one-way transport index (R), overall moisture management capacity (OMMC).

### 3.1. Liquid Moisture Transport of Knitted Fabrics for Underwear

The liquid moisture transport properties of four types of knitted fabrics intended for firefighter underwear are presented in Table 3 and Table 4 [27].

Among all the parameters provided by the MMT instrument, two are the most important: accumulative one-way transport index (R) and Overall Moisture Management Capacity (OMMC). Both parameters describe the sample as a whole, while the remaining parameters determined by the MMT pertain to specific sides of the sample. Both R and OMMC are calculated based on other parameters measured by the device, according to the algorithms developed by the MMT manufacturer.

The R parameter represents the difference between the accumulative moisture content of the bottom surface and the top surface relative to time [21]. The interpretation is as follows: a higher value of the R parameter indicates better liquid moisture transport and, consequently, better moisture management [21,26].

The Overall Moisture Management Capacity (OMMC) is a parameter indicating the overall ability of the measured material to transport moisture in a liquid state. Generally, the OMMC is calculated from the values of the absorption rate for the bottom surface (BAR), the spreading speed for the bottom surface (SSB), and the accumulative one-way transport index (R). The value of the OMMC parameter ranges from 0 to 1. A higher OMMC value indicates a better ability of the measured specimen to transport liquid moisture. Based on the OMMC value, fabrics are classified into five categories, ranging from very poor (OMMC = 0.0–0.2) to excellent (OMMC > 0.8) [22].

The Overall Moisture Management Capacity (OMMC) of the knitted fabrics being tested ranges from 0.12 to 0.76 (Table 4). Based on these values, it can be concluded that the best performance in terms of liquid moisture transport is observed for the F3 knitted fabric variant (OMMC = 0.76). This fabric is a single jersey made of a 54% cotton and 46% polyester blend. The lowest OMMC value (0.12) was recorded for the F1 knitted fabric, a special fabric made from a modacrylic/cotton/polyamide blend. Intermediate results for the OMMC parameter were observed for the F2 and F1 knitted fabrics, which is further confirmed by the R parameter values presented in Table 4.

The superior performance of the F3 fabric can be attributed to several key factors. This fabric demonstrates the longest wetting time for the top surface (WTT = 90.66 s), indicating that moisture is absorbed slowly by the surface in contact with the user’s skin. Additionally, the absorption rate for the top surface (TAR = 22.60%/s) is significantly lower than that of the other fabric variants. These factors contribute to a positive physiological comfort experience by minimizing the moisture retained near the skin. Furthermore, the F3 fabric exhibits an excellent spreading speed on the bottom surface (SSB = 2.06 mm/s), which is much higher than that of the other fabrics. This enhances moisture distribution across the surface, promoting faster evaporation. The maximum wetted radius on the bottom surface (MWRB = 10 mm), which is also the largest among the tested fabrics, further facilitates efficient moisture management and rapid drying. Together, these characteristics ensure that the F3 fabric not only keeps the skin dry but also promotes effective moisture transport and evaporation, contributing to both comfort and performance.

Thus, we can evaluate the tested knitted fabrics as if they are used on their own, with their outer layer in contact with the environment. In the case of underwear worn under the FPC, the outer surface adheres to the inner surface of the FPC. This influences liquid moisture transport both in the individual underwear fabric and in the assembly consisting of the underwear fabric and the multilayer textile set applied in the FPC.

### 3.2. Liquid Moisture Transport in the Multilayer Textile Sets for the FPC

The investigations presented aim to assess the influence of the composition of the entire outfit, consisting of underwear and the FPC, on the liquid transport parameters of the firefighter’s outfit. To this end, four multilayer textile sets were also measured for liquid moisture transport. The measurements showed that the investigated multilayer sets are impermeable to liquid moisture. In all cases, no liquid was visible on the outer layer of the sets [8]. The values of the parameters for the bottom (outer) surface of the sets—such as absorption rate, spreading speed, maximum wetted radius, and overall moisture management capacity—were all equal to 0. The wetting time for the bottom surface was 120 s, which corresponds to the test duration. This indicates that, during the test, the outer surface of the measured sets did not become wet. All these results confirm that liquid moisture cannot pass through the entire multilayer textile sets intended for the FPC. Analysis of the samples after the test revealed that moisture traces were observed only on the first and second layers of the multilayer textile sets. Therefore, in the next step of the investigations, the first (inner) and second (middle) layers were measured separately. The middle layer was also assessed as impermeable to liquid water, which is expected since it serves as a moisture barrier. Measurements showed that only the inner layer of the multilayer textile sets facilitates the transfer of liquid moisture. The results of the measurements for the inner layer of the multilayer packages are presented in Table 5 and Table 6 [27].

Based on the presented results, it is justified to state that the inner layers of the multilayer textile sets intended for the FPC differ from each other in all the parameters measured by the MMT. However, for both calculated parameters—R and OMMC—all investigated inner layers of the multilayer sets were assessed as poor or very poor in terms of liquid transport. In all cases, the value of the R parameter (accumulative one-way transport index) was negative (Table 6). This indicates that the materials used for the inner layer of the multilayer sets for the FPC are not effective in terms of liquid sweat evaporation and the physiological comfort of the clothing wearer.

Among all the investigated fabrics forming the inner layer of the multilayer sets for the FPC, only the SS1 variant can be classified as poor (OMMC = 0.21). However, the value of the OMMC parameter is practically at the boundary between the very poor and poor classes. The remaining investigated materials were classified as very poor, with the OMMC value for the SS1 variant being very close to the threshold between the very poor and poor classes.

The values of other parameters provided by the MMT confirm the poor moisture wicking efficiency of the investigated inner layers of the multilayer sets intended for the FPC. For example, the maximum wetted radius of the top surface of the inner layers in the S2, S3, and S4 sets is very high, indicating a large area of moisture trace on the inner surface. When using these materials directly next to the skin, a large area of moisture trace should be considered a disadvantage from the perspective of physiological comfort. However, it is important to note that these materials are worn together with underwear, and a large moisture trace area can be advantageous, as it suggests that the inner layer of the multilayer set can absorb moisture from the underwear efficiently. Furthermore, the large moisture trace on the bottom surface can be seen as beneficial, as it means that the inner layer of the multilayer set transports moisture well from the inner to the outer surface, thereby allowing moisture to be carried further away from the skin compared to when a small moisture trace is observed on the outer side of the material.

In a similar way, the other parameters provided by the MMT for the inner layers of the multilayer textile sets for the FPC can be discussed. However, the aim of this work was to analyze the impact of the composition of assemblies containing both the underwear and FPC on liquid moisture transport. Since only the first (inner) layer of the FPC transports liquid moisture, the next step of the investigations involved creating assemblies for measurement that combined the knitted fabrics for underwear with the inner layers of the multilayer textile sets intended for the FPC.

### 3.3. Liquid Moisture Transport in Assemblies Composed of Knitted Fabrics for Underwear and Inner Middle Layer of Multilayer Sets for the FPC

A total of 16 variants of assemblies composed of knitted fabrics for underwear and the inner (thermal) layer of the textile sets for the FPC were created and measured using the MMT.

Based on the results, it was concluded that in all cases, the variant of the multilayer set from which the inner layer was taken significantly influences the values of the parameters from the MMT at the significance level of 0.05. In the case of the knitted fabric variant, only for the following parameters: BAR, MWRT, and SST, was the influence of the knitted fabric variant statistically significant.

**Figure 3:**
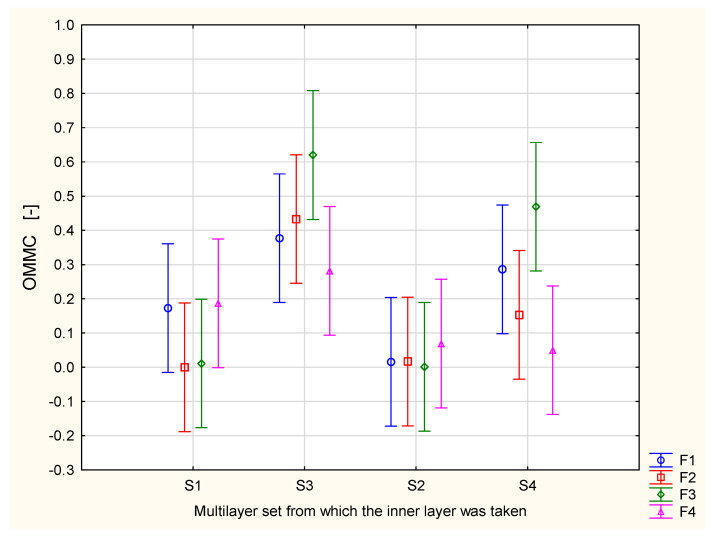
Overall Moisture Management Capacity (OMMC) of the assemblies created from the knitted fabric and the inner layer of the multilayer set.

The results of the OMMC measurement are presented in Figure 3. It is clearly observed that the highest values occurred for the assemblies containing the inner layer from the S3 multilayer set. However, when measuring only the inner layers individually, the inner (top) layer from the S3 multilayer set was rated worse in terms of the OMMC parameter than the inner layers of the S1 and S4 packages (Table 6). The same situation is observed in the case of the knitted fabrics. When measuring only the knitted fabrics (Table 4), the F3 fabric was assessed as the best one based on the OMMC values. In assemblies, the relationships have changed. Only in combination with the inner layer of the S3 and S4 multilayer sets does the F3 knitted fabric for underwear provide the best performance—the highest values of the OMMC parameter among all investigated assemblies. When the F3 knitted fabric is combined with the inner layer from the S1 and S2 multilayer sets, the OMMC parameter values are lower than those for the assemblies created using other knitted fabrics.

The results for the assemblies created using the F4 knitted fabric are particularly interesting. The F4 fabric was assessed as the worst among all the investigated knitted fabrics (Table 4). While selecting materials for firefighter underwear, it could be rejected due to its poor sweat transport performance, although it is specifically designed for firefighters. However, when combined with the inner layer from the S1 and S3 multilayer sets, the highest values of the OMMC parameter were obtained among all assemblies created with the inner layers from these two multilayer sets (Figure 3).

**Figure 4:**
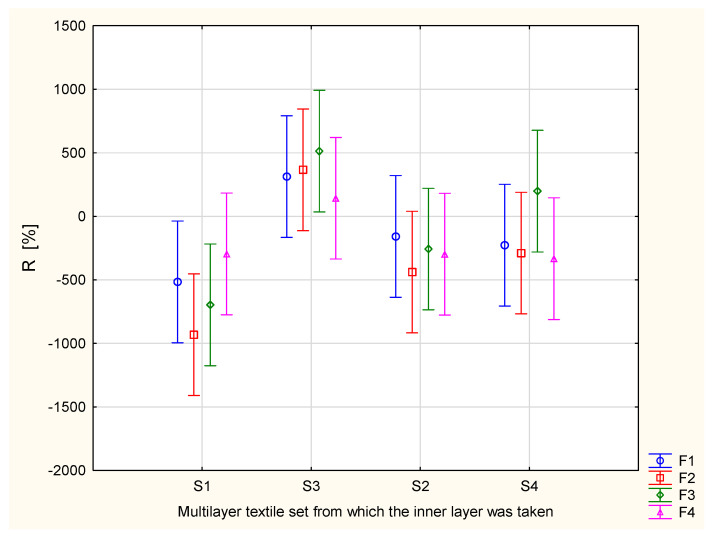
Accumulative one-way transport index of the assemblies created from the knitted fabric and inner layer of the multilayer set.

Similar relationships were observed for the R parameter—the accumulative one-way transport index (Figure 4).

Measurements showed that the entire multilayer textile sets intended for firefighters’ protective clothing, as well as the middle layer of these multilayer sets, do not transfer liquid moisture (sweat). This means that the transport of liquid moisture from the firefighter’s body stops at the middle layer, leaving the liquid sweat trapped between the firefighter’s skin and the outer layer of their outfit. Therefore, efforts should be made to ensure that most of the sweat condensed on the firefighter’s skin is transferred as far away from the skin as possible, since it cannot be completely removed to the outside. In this context, the maximum wetted radius for the top surface of the assemblies may be an important indicator for assessing the multilayer assemblies of the firefighter’s outfit. The smaller the maximum wetted radius on the inner surface is, the smaller the wet area on the inner side of the underwear adjacent to the firefighter’s skin. This, in turn, means better wicking of sweat from the surface adjacent to the skin, helping to minimize the discomfort caused by contact with wet underwear.

**Figure 5:**
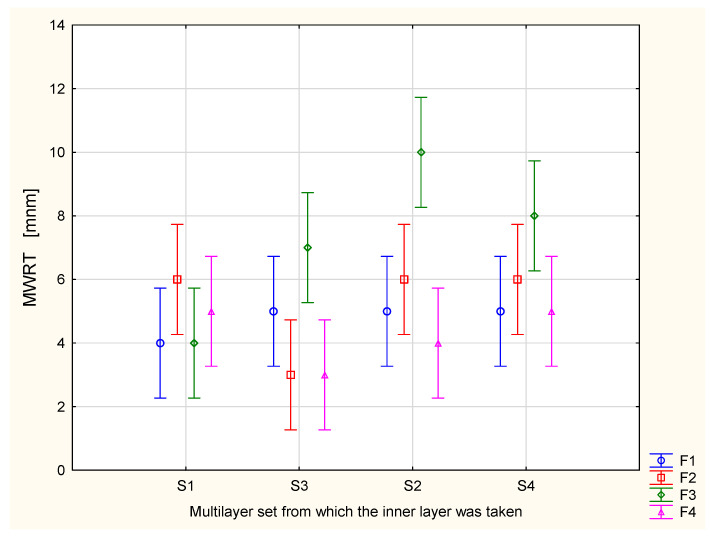
Maximum wetted radius of the top surface of the assemblies created from the knitted fabric and inner layer of the multilayer set.

Figure 5 shows the values of the maximum wetted radius for the top surface of the investigated assemblies containing the knitted fabric for underwear and the inner layer from the multilayer sets. It is clearly seen that by combining the same underwear with different multilayer sets, different values of the maximum wetted radius for the top surface can be obtained. The F3 knitted fabric, which was individually assessed as the best among the investigated knitted fabrics for underwear, is characterized by the smallest value of the MWRT parameter when combined with the S1 multilayer set. In contrast, the F4 knitted fabric, which was rated as the worst among the other knitted fabrics in terms of liquid sweat transport, shows the smallest value of the maximum wetted radius of the top surface when combined with the inner layers from the S2, S3, and S4 assemblies. This indicates the best performance in terms of wicking liquid sweat away from the skin.

It is not easy to decide which assembly is the best from the point of view of liquid moisture transport. Liquid moisture transport is a complex phenomenon, resulting from several factors characterized by the particular parameters provided by the MMT. According to the OMMC parameter values, the best performance was observed for the S3 + F3 assembly, followed by S4 + F3, then S3 + F2, and S3 + F1. Considering the R parameter, the best performance was observed for the following assemblies: S3 + F3, followed by S3 + F2, S3 + F1, S4 + F3, and S3 + F4. When analyzing the MWRT (maximum wetted radius for the top surface) parameter, a lower value is better from the point of view of physiological comfort. According to Figure 5, the lowest values of MWRT were observed for the S3 + F2 and S3 + F4 assemblies. Taking this into consideration, the S3 + F2 assembly seems to be the best option to optimize liquid moisture transport in the assemblies. The S3 + F4 variant is also worth mentioning. It contains the F4 knitted fabric, which is made of a blend of cotton, polyamide, modacrylic, and antistatic fibers (Table 1). The F4 fabric has the additional advantages of ensuring nonflammability and antistatic properties. Together with the S3 multilayer set, the F4 knitted fabric may not provide the best performance but still offers very good liquid transport performance.

It should be noted that the assessment of the assemblies based on the OMMC and R parameters differs from that based on the MWRT parameter. The R and OMMC are synthetic parameters calculated from the parameters determined for the individual surfaces of the investigated sample, whereas the other parameters directly characterize particular phenomena: sweat absorption, spreading, and transfer from the top to the bottom surface. In the case of complex materials such as the multilayer assemblies for firefighters’ outfits, and considering that liquid sweat remains between the layers of the outfit, it is essential to determine which parameters are most crucial for ensuring the physiological comfort of the firefighter during action. In our opinion, further investigations are needed, possibly in conjunction with utility trials of firefighter outfits composed of different underwear and FPC combinations.

## 4. Conclusions

For the physiological comfort of a firefighter during rescue operations, the transport of sweat produced by the body is crucial. The underwear is the layer next to the skin in the firefighter’s outfit, in direct contact with the skin and the liquid sweat condensed on it. However, the underwear is worn together with the firefighter’s multilayer protective clothing, where each layer creates a barrier for water vapor. Under intensive sweating conditions, some of the sweat produced by the firefighter’s body is not evaporated from the skin into the environment but condenses on the skin surface, leading to unpleasant feelings and discomfort. In such situations, the transport of sweat in its liquid form is essential to ensure physiological comfort and optimal effectiveness of firefighters in action.

Measurements of the entire multilayer textile sets intended for the FPC, regarding liquid moisture transport, showed that no liquid moisture was observed on the outer layer of the multilayer sets. Liquid moisture was only observed in the inner layer of the sets. This indicates that the liquid moisture remains inside the multilayer fabric sets and accumulates during the use of the firefighter’s protective garment.

Underwear, typically made of knitted fabrics, generally transports liquid moisture well. Measuring these knitted fabrics using the Moisture Management Tester allows for the assessment of their liquid sweat transport performance and helps with selecting the best one in this regard. However, measurements of assemblies composed of underwear fabrics combined with the inner layer of multilayer textile sets for the FPC showed that the liquid transport performance of such assemblies depends on their composition. Connecting the same knitted fabric (the same underwear) with different FPC layers results in different values for the parameters characterizing liquid moisture transport. The combination of the investigated knitted fabrics with the inner layer of specific multilayer sets alters the relationship between the knitted fabrics in terms of their liquid transport performance. This leads to the conclusion that the liquid transport performance of the entire firefighter’s outfit can be optimized by appropriately selecting both the underwear and the inner layer of the multilayer set for the FPC. Therefore, it is necessary to measure liquid moisture transport using the MMT not only for individual components of the outfit but also for assemblies composed of individual components such as underwear and the inner layer of the multilayer set for the FPC.

Among the created and measured variants of multilayer assemblies for the firefighter’s outfit, the best performance from the liquid moisture transport perspective was observed for the assembly containing the S3 multilayer set for the FPC and the F2 knitted underwear. The liquid sweat transport performance of the S3 + F4 assembly is slightly worse than that of the S3 + F2 assembly, but it offers additional advantages. It contains the F4 knitted fabric, which is specifically designed for firefighters and provides added functionality, such as flame retardance and electroconductivity.

## Figures and Tables

**Table 1:** Characteristic parameters of the investigated knitted fabrics.

Fabric Variant	Stitch	Fiber Composition	Thickness	Mass per Square Meter g/m^2^	Wale Density Wale/cm	Course Density Course/cm
F1	Single jersey	cotton	0.45	161.09	15	22
F2	Pique	97% cotton/ 3% elastane	0.71	138.59	10	12
F3	Single jersey	54% cotton/ 46% polyester	0.66	205.47	11	16
F4	Single jersey	51% modacrylic, 26% cotton, 19% polyamide, 2% antistatic fiber 1% elastane	0.67	182.50	16	22

**Table 2:** Characteristics of multilayer textile sets intended for the FPC.

Multilayer Set Variant	Layer	A Kind of Material	Layer Connection	Raw Material Composition	Mass per Square Meter g/m^2^	Thickness mm
S1	Outer shell	Woven fabric	-	99 aramid/1 ASF	210	0.50
	Moisture barrier	Knitted fabric laminated	-	PES PU laminate	80	0.25
	Thermal barrier Lining	Nonwoven Woven fabric	-	aramid cotton FR	275	1.24
S2	Outer shell	Woven fabric	-	98 meta-aramid/ASF	210	0.47
	Moisture barrier	Laminate/nonwoven	-	PTFE/aramid	165	1.08
	Thermal barrier Lining	Felt Woven fabric	Quilted	aramid 50 aramid/50 VI FR	125 50	0.80
S3	Outer shell	Woven fabric	-	58 para-aramid/40 PBI/2 ASF	205	0.40
	Moisture barrier	Laminate/nonwoven	-	PTFE/aramid	100	0.47
	Thermal barrier (lining and felt)	Nonwoven	Quilted	aramid	170	0.73
		Woven fabric				
S4	Outer shell	Woven fabric	-	99 aramid/1 ASF	210	0.46
	Moisture barrier	Laminate	-	65 aramid/35 PU	120	0.76
	Thermal barrier	Knitted fabric	Quilted	aramid + FR	270	1.33
	Lining	Woven fabric		50 aramid/50 VI		

Legend: PES—polyester, PU—polyurethane, FR—flame retardant, ASF—antistatic fiber, PTFE—Polytetrafluoroethylene, VI—viscose.

**Table 3:** Liquid moisture transport parameters of knitted fabrics.

Fabric Variant		WTT (s)	WTB (s)	TAR (%/s)	BAR (%/s)	MWRT (mm)	MWRB (mm)
F1	Mean	55.53	6.48	245.49	50.85	3.00	5.00
	SD	58.89	1.95	232.31	8.65	2.74	0.00
	CV	1.06	0.30	0.95	0.17	0.91	0.00
F2	Mean	32.67	76.90	351.06	65.04	4.00	2.00
	SD	48.89	59.33	209.24	114.66	2.24	2.74
	CV	1.50	0.77	0.60	1.76	0.56	1.37
F3	Mean	90.66	8.76	22.68	73.88	2.00	10.00
	SD	46.16	2.56	44.47	30.45	2.74	0.00
	CV	0.51	0.29	1.96	0.41	1.37	0.00
F4	Mean	33.64	97.16	393.73	10.85	4.00	1.00
	SD	48.28	51.07	233.47	24.26	2.24	2.24
	CV	1.44	0.53	0.59	2.24	0.56	2.24

Legend: SD—standard deviation, CV—variation coefficient.

**Table 4:** Liquid moisture transport parameters of knitted fabrics, continuation.

Fabric Variant		SST (mm/s)	SSB (mm/s)	R (%)	OMMC (-)
F1	Mean	0.24	0.80	424.31	0.41
	SD	0.23	0.24	523.47	0.24
	CV	0.94	0.30	1.23	0.59
F2	Mean	0.39	0.31	−370.51	0.18
	SD	0.25	0.58	860.32	0.29
	CV	0.65	1.84	2.32	1.59
F3	Mean	0.08	2.06	1021.32	0.76
	SD	0.15	0.50	193.55	0.10
	CV	1.83	0.24	0.19	0.14
F4	Mean	0.33	0.17	−551.04	0.12
	SD	0.18	0.37	786.12	0.28
	CV	0.56	2.24	1.43	2.24

Legend: SD—standard deviation, CV—variation coefficient.

**Table 5:** Liquid moisture transport parameters of the inner layer of multilayer textile sets for firefighter protective clothing.

Variant of Multilayer Set		WTT (s)	WTB (s)	TAR (%/s)	BAR (%/s)	MWRT (mm)	MWRB (mm)
S1 inner layer	Mean	12.04	39.31	394.39	22.48	7.00	9.00
	SD	3.71	47.34	216.43	18.93	2.74	5.48
	CV	0.31	1.20	0.55	0.84	0.39	0.61
S2 inner layer	Mean	2.30	60.88	75.89	4.26	30.00	3.00
	SD	0.47	54.38	3.87	4.32	0.00	4.47
	CV	0.20	0.89	0.05	1.01	0.00	1.49
S3 inner layer	Mean	2.64	37.98	71.74	24.50	29.00	11.00
	SD	1.44	45.71	20.14	20.57	2.24	9.62
	CV	0.55	1.20	0.28	0.84	0.08	0.87
S4 inner layer	Mean	2.73	6.40	62.34	40.45	30.00	16.00
	SD	0.91	1.15	6.61	6.37	0.00	2.24
	CV	0.33	0.18	0.11	0.16	0.00	0.14

**Table 6:** Liquid moisture transport parameters of the inner layer of multilayer textile sets for firefighter protective clothing, continued.

Sample		SST (mm/s)	SSB (mm/s)	R (%)	OMMC (-)
S1 inner layer	Mean	0.48	0.57	−168.88	0.18
	SD	0.16	0.37	594.09	0.20
	CV	0.34	0.65	3.52	1.10
S2 inner layer	Mean	7.76	0.37	−1026.4	0.01
	SD	1.88	0.63	37.44	0.02
	CV	0.24	1.70	0.04	2.24
S3 inner layer	Mean	13.41	0.97	−849.57	0.08
	SD	46.11	1.10	736.61	0.07
	CV	3.44	1.14	0.87	0.87
S4 inner layer	Mean	5.07	2.51	−598.44	0.21
	SD	0.98	0.48	58.13	0.03
	CV	0.19	0.19	0.10	0.14

## Data Availability

The original contributions presented in the study are included in the article, further inquiries can be directed to the corresponding author.
